# Association of Body Mass Index With Disability in Activities of Daily Living Among Chinese Adults 80 Years of Age or Older

**DOI:** 10.1001/jamanetworkopen.2018.1915

**Published:** 2018-09-07

**Authors:** Yue-Bin Lv, Jin-Qiu Yuan, Chen Mao, Xiang Gao, Zhao-Xue Yin, Virginia Byers Kraus, Jie-Si Luo, Hua-Shuai Chen, Yi Zeng, Wen-Tao Wang, Jiao-Nan Wang, Xiao-Ming Shi

**Affiliations:** 1 National Institute of Environmental Health, Chinese Center for Disease Control and Prevention, Beijing, China; 2 Division of Epidemiology, Jockey Club School of Public Health and Primary Care, Chinese University of Hong Kong, Hong Kong, China; 3 Department of Epidemiology, School of Public Health, Southern Medical University, Guangzhou, Guangdong, China; 4 Department of Nutritional Sciences, Pennsylvania State University, Philadelphia; 5 Division of Non-Communicable Disease Control and Community Health, Chinese Center for Disease Control and Prevention, Beijing, China; 6 Duke Molecular Physiology Institute and Division of Rheumatology, Department of Medicine, Duke University School of Medicine, Durham, North Carolina; 7 Center for the Study of Aging and Human Development, Geriatric Division of School of Medicine, Duke University, Durham, North Carolina; 8 Center for Study of Healthy Aging and Development Studies, Raissun Institute for Advanced Studies, Peking University, Beijing, China

## Abstract

**Importance:**

Body mass index (BMI) shows a U-shaped association with impaired physical functioning among adults; the association is reduced or eliminated with aging.

**Objective:**

To examine whether BMI is associated with subsequent disability in activities of daily living (ADL) in Chinese adults age 80 years or older.

**Design, Setting, and Participants:**

Data were obtained on 16 022 adults age 80 years or older who were able to perform ADL independently at baseline from the Chinese Longitudinal Healthy Longevity Study, a community-based prospective cohort study conducted in 23 provinces of China. The study was initiated in 1998, with follow-up and recruitment of new participants in 2000, 2002, 2005, 2008, 2011, and 2014.

**Main Outcomes and Measures:**

Disability in ADL was defined as dependence in eating, toileting, bathing, dressing, indoor activities, and/or continence.

**Results:**

Among the 16 022 participants, 45.2% were men and 54.8% were women, with a mean (SD) age of 92.2 (7.2) years and a mean (SD) BMI (calculated as weight in kilograms divided by height in meters squared) of 19.3 (3.8). During 70 606 person-years of follow-up, 8113 participants with disability in ADL were identified. Cox proportional hazards regression models with penalized splines showed that BMI was linearly associated with disability in ADL: each 1-kg/m^2^ increase in BMI corresponded to a 4.5% decrease in the risk of disability in ADL. In comparison with individuals in the fourth quintile for BMI, the adjusted hazard ratio for disability in ADL was 1.38 (95% CI, 1.29-1.48) in the first quintile, 1.37 (95% CI, 1.28-1.47) in the second quintile, 1.11 (95% CI, 1.04-1.19) in the third quintile, and 0.85 (95% CI, 0.79-0.91) in the fifth quintile (*P* < .001 for trend). When BMI was categorized by Chinese guidelines, the underweight group (BMI <18.5) showed significantly increased risk of disability in ADL (hazard ratio, 1.34; 95% CI, 1.28-1.41) and the overweight or obese group (BMI ≥24.0) showed significantly decreased risk of disability in ADL (hazard ratio, 0.84; 95% CI, 0.78-0.91) compared with the normal weight group (BMI 18.5 to <24.0) (*P* < .001 for trend).

**Conclusions and Relevance:**

Higher BMI was associated with a lower risk of disability in ADL among Chinese adults age 80 years or older, which suggests that current recommendations for BMI may need to be revisited. More attention should be paid on underweight, rather than overweight or obesity, for the prevention of disability in ADL after age 80 years.

## Key Points

**Question:**

Is higher body mass index or overweight or obesity associated with higher risk of subsequent disability in activities of daily living in Chinese adults 80 years of age or older?

**Findings:**

In this cohort study of 16 022 Chinese adults aged 80 years or older, body mass index was linearly and inversely associated with disability in activities of daily living. When body mass index was categorized by quintiles or categorized by Chinese guidelines, higher body mass index, including being overweight or obese, showed significantly decreased risk of disability in activities of daily living.

**Meaning:**

The finding suggests that current recommendations for body mass index may need to be revisited; more attention should be paid to underweight rather than overweight or obesity for the prevention of disability in activities of daily living in adults 80 years of age or older.

## Teaser

This cohort study uses data from the Chinese Longitudinal Healthy Longevity Study to examine whether body mass index (BMI) is associated with subsequent disability in activities of daily living in Chinese adults 80 years of age or older.

## Introduction

A marked increase has been documented in the prevalence worldwide, including China, of individuals with overweight and obesity, usually assessed by body mass index (BMI; calculated as weight in kilograms divided by height in meters squared).^1,2^ Individuals with overweight and obesity constitute a major public health problem owing to the increased risk of hypertension, dyslipidemia, coronary heart disease, stroke, type 2 diabetes, musculoskeletal disorders, and some kinds of cancer.^3^ Body mass index shows a U-shaped association with morbidity, impaired physical functioning, and mortality among adults. However, a consistent and puzzling finding is the reduction or elimination of this association with increasing age.^4^ Previous research and discussions have focused on the association of increased BMI with morbidity and mortality for older adults, but associations of BMI with many important measures of health, such as disability in activities of daily living (ADL), have not been fully examined.

Accurate quantification of the role of BMI (underweight, normal weight, overweight, or obesity) in the incidence of disability in ADL is desirable in the face of the increasing prevalence of individuals with overweight and obesity, and prolonged life span in the population. Disability in ADL is considered the most serious form of disability measure^5^; it is defined as difficulty undertaking activities in any areas of daily life due to a health issue or a physical problem.^6^ At some point, a person with disability in ADL may be unable to live independently and may require assistance from family or institutionalization. Approaches to measuring disability vary across countries. Independent of how disability in ADL was measured, many studies have addressed the association between BMI and disability in ADL at older ages: both longitudinal^7,8,9^ and cross-sectional^10,11,12,13^ studies have consistently found that obesity was an independent risk factor for disability in ADL in older adults.

However, little is known regarding the association between BMI and disability among adults age 80 years or older, the age group most susceptible to disability, which is a leading indicator of health status and major determinant of quality of life for the adults in this age group.^14^ A recent large longitudinal study showed that obesity at baseline was an important and potentially modifiable risk factor for incident functional disability before age 85 years in older women.^15^ Although 2 cross-sectional studies have shown associations of obesity with poor physical functioning,^16,17^ longitudinal studies have shown that individuals with a BMI of 25 or greater are likely to have better functional performance.^18,19^ Potential limitations of the previous studies of the oldest old include small sample size (<1000 participants), cross-sectional study design,^17^ oversampling of octogenarians, ascertainment of BMI by self-report, and varying measures of disability. We attempted to overcome these limitations in this study by examining the association of objectively measured BMI and internationally used measures of disability in ADL among 16 022 Chinese adults 80 years of age or older during 70 606 person-years of follow-up.

## Methods

### Study Participants

This study analyzed data obtained from the Chinese Longitudinal Healthy Longevity Study (CLHLS), a prospective cohort study conducted in 23 provinces of China. The CLHLS has the largest samples of adults age 80 years or older in the world; it was initiated in 1998, with subsequent follow-up and recruitment of new participants in 2000, 2002, 2005, 2008, 2011, and 2014. The study was the first national longitudinal survey on determinants of healthy aging among the oldest old individuals in China. More detailed descriptions of the CLHLS have been reported in previous studies.^17,20^ This study was approved by the Medical Ethics Committee of Peking University. All participants or their legal representatives signed written consent forms to participate in the baseline and follow-up surveys. This study followed the Strengthening the Reporting of Observational Studies in Epidemiology (STROBE) reporting guidelines.

Among the 43 487 participants in 7 waves of CLHLS, those excluded were 7128 participants age 79 years or younger, 5019 participants without BMI data, 3501 participants lost to follow-up at the first follow-up survey, and 11 817 participants with baseline disability in ADL. Accordingly, the final sample that met inclusion criteria (age ≥80 years, normal baseline ADL, and available BMI data) for this study was 16 022 participants (eFigure 1 in the Supplement). The sample consisted of 7243 women and 8779 men; 6281 participants were 80 to 89 years of age, 6210 were 90 to 99 years of age, and 3531 were 100 years of age or older. To test the possibility of potential selection bias, BMI, age, and sex were compared between participants lost to follow-up (5019 participants) or not (27 839 participants) at the first follow-up survey; a significant difference was found for BMI (19.6 vs 19.2) and age (93.1 vs 94.0 years) between the 2 groups, while there was no significant difference for sex (38.2% vs 39.2% were men).

### Measurement and Calculation of BMI

In the physical examination, body weight and height were measured by trained medical staff using a standardized protocol. Body weight was measured to the nearest 1 kg for individuals wearing light clothing. Height was measured to the nearest 1 cm in the 2005, 2008, and 2011 surveys, or was estimated on the basis of knee height (vertical distance from the sole of the foot to the upper surface of the knee, with the knee and ankle each flexed to a 90° angle) using a validated equation in the 1998, 2000, and 2002 surveys (men, height = 67.78 + 2.01 × knee height; women, height = 74.08 + 1.81 × knee height).^21^ Body mass index was divided into 4 categories according to the guideline for Chinese individuals: underweight (BMI <18.5), normal weight (BMI 18.5 to <24.0), overweight (BMI 24.0 to <28.0), and obese (BMI ≥28.0).^22^ Given that only 2.5% of the participants were defined as obese, those participants were combined with the participants defined as overweight. Quintiles of BMI (the first, second, third, fourth, and fifth quintiles) were created for further analyses; the corresponding cutoffs were less than 16.2 for the first quintile, 16.2 to 17.9 for the second quintile, 18.0 to 19.8 for the third quintile, 19.9 to 22.1 for the fourth quintile, and 22.2 or more for the fifth quintile.

### Measurement of Disability

Disability in ADL was assessed using the Katz Index of Independence in ADL.^23^ This scale includes 6 tasks performed by individuals in daily life that are essential to independent living: eating, toileting, bathing, dressing, indoor activities, and continence.^24^ Each item is scored 0 or 1, where 0 indicates the inability to perform the task independently, and 1 indicates the ability to perform the task independently. Total scores ranged from 0 to 6, with higher scores indicating better ADL. Disability in ADL was defined as inability to perform any task independently; participants were considered as having independent ADL if they were able to perform all tasks independently. The CLHLS assessed disability in ADL every 2 or 3 years from baseline to 2014 using the Katz Index of Independence in ADL for participants still living (answered by the participants themselves) and decedents of those who had died (answered by closest relatives) to document the incidence of disability in ADL for participants during the follow-up survey; this method prevented censoring of decedents on the basis of lack of ADL data owing to mortality.

### Potential Confounders

Data on potential confounders were collected and defined as follows: sociodemographic information such as age (as a continuous variable), sex (men or women), residence (urban or rural), educational background (literacy, receiving >1 year of any formal education; and illiteracy, receiving <1 year of formal education), current marital status (married or not), and living pattern (living with family members or not); lifestyle behaviors including smoking status (current smoker, former smoker, or nonsmoker), alcohol consumption status (current drinker, former drinker, or nondrinker), and regular exercise (yes or no); prevalence of heart disease (yes or no), cerebrovascular disease (yes or no), type 2 diabetes (yes or no), and respiratory disease (yes or no); and systolic blood pressure and diastolic blood pressure, measured with a mercury sphygmomanometer by trained internists. Hypertension was defined as systolic blood pressure of 140 mm Hg or higher and/or diastolic blood pressure of 90 mm Hg or higher, or self-report of a diagnosis of hypertension by a physician.

### Statistical Analysis

On the whole, the missing data for any individual covariates amounted to less than 0.7%; thus, multiple imputation methods were performed for the correction of missing values on the individual covariates. The Cochran-Armitage test for trends was performed to compare the difference for categorical variables. Analysis of variance for trends was performed to compare the difference for continuous variables among participants with different BMI quintiles.

Time to disability in ADL (event = 1) was defined as the period from baseline to the first time a participant experienced disability. For those who died without disability in ADL (event = 2), the time from baseline to time of death was calculated. Censored (event = 0) observations were defined as participants who did not experience disability in ADL; the censoring time was calculated from baseline to the last assessment of ADL tasks or loss to follow-up (eFigure 1 in the Supplement). Cox proportional hazards regression assumption was tested with Kaplan-Meier curves when BMI was taken as a categorized variable, then was tested by the linear regression of the scaled Schoenfeld residuals on functions of time when BMI was taken as a continuous variable (eAppendix in the Supplement). It showed that the Cox proportional hazards regression assumption was satisfied (eFigures 2-4 in the Supplement).^25^ Cox proportional hazards regression models with penalized splines were conducted to examine the linear or nonlinear association of BMI with disability, using BMI as a continuous variable. Because the incidence of disability in ADL may be superseded by the incidence of mortality, competing risk models were used to explore the association of BMI with disability, accounting for major identified risk factors of disability in ADL to estimate the hazard ratio (HR) and 95% CIs, with the fourth BMI quintile (normal weight) being the reference group. Age was treated as a continuous variable; categorical variables in the adjusted models were sex, residence, living pattern, current marital status, educational background, alcohol consumption practice, smoking practice, regular exercise, hypertension, heart disease, type 2 diabetes, cerebrovascular disease, and respiratory disease.

In the further analyses, the following sensitivity analyses were conducted: (1) separately in participants 80 to 89 years of age, 90 to 99 years of age, and 100 years of age or older, to explore the difference in the 3 age groups; (2) separately for men and women, on the basis of the assumption that the association of disability in ADL with BMI was sex dependent^15^; (3) for a “healthy” group without identified comorbidities (diabetes, heart disease, cerebrovascular disease, or respiratory disease), to assess whether the findings were affected by inclusion of participants with lower BMI and poorer health for other reasons; (4) in the participants who were former smokers or nonsmokers, to assess whether the association of BMI and disability in ADL was modified by smoking status (based on a previous finding^26^ of a stronger association between higher BMI and mortality in nonsmokers); and (5) exploring interactions of age, sex, comorbidities, and smoking status with BMI for the incidence of disability in ADL.

Analyses were performed with SAS, version 9.4 (SAS Institute Inc), and R, version 3.4.2 (R Foundation for Statistical Computing) was used to perform Cox proportional hazards regression models with penalized splines. All *P* values were from 2-sided tests and results were deemed statistically significant at *P* < .05 for all analyses.

## Results

Among the 16 022 participants, 45.2% were men and 54.8% were women, with a mean (SD) age of 92.2 (7.2) years. The mean (SD) BMI of the 16 022 participants was 19.3 (3.8). The prevalence of obesity was 2.5%, and the prevalence of overweight was 9.6%. As shown in the Table, the participants with higher BMI were more likely to be younger, be men, live in urban areas, live with family members, be literate, be married, be nonsmokers, engage in regular exercise, have hypertension, have a history of type 2 diabetes, have a history of heart disease, have a history of cerebrovascular disease, and have normal cognition.

**Table.** Baseline Characteristics of Chinese Adults 80 Years of Age or Older by Quintiles of Body Mass Index

Characteristics	Chinese Adults, No. (%)						*P* Value
	First Quintile	Second Quintile	Third Quintile	Fourth Quintile	Fifth Quintile	All	
No. of participants	3266 (20.4)	3072 (19.2)	3299 (20.6)	3137 (19.6)	3248 (20.3)	16 022 (100)	
Age, mean (SD), y	94.0 (7.3)	93.0 (7.1)	92.3 (7.1)	91.4 (7.1)	90.2 (6.9)	92.2 (7.2)	<.001
Sex							
Male	1072 (32.8)	1290 (42.0)	1552 (47.0)	1589 (50.7)	1740 (53.6)	7243 (45.2)	<.001
Female	2194 (67.2)	1782 (58.0)	1747 (53.0)	1548 (49.3)	1508 (46.4)	8779 (54.8)	
Residence							
Urban	415 (12.7)	466 (15.2)	620 (18.8)	689 (22.0)	852 (26.2)	3042 (19.0)	<.001
Rural	2851 (87.3)	2606 (84.8)	2679 (81.2)	2448 (78.0)	2396 (73.8)	12 980 (81.0)	
Marital status							
Married	529 (16.2)	637 (20.7)	769 (23.3)	902 (28.8)	1118 (34.4)	3955 (24.7)	<.001
Unmarried	2737 (83.8)	2435 (79.3)	2530 (76.7)	2235 (71.2)	2130 (65.6)	12 067 (75.3)	
Educational background^a^							
Illiteracy	2477 (75.8)	2199 (71.6)	2194 (66.5)	2000 (63.8)	1889 (58.2)	10 759 (67.2)	<.001
Literacy	789 (24.2)	873 (28.4)	1105 (33.5)	1137 (36.2)	1359 (41.8)	5263 (32.8)	
Living pattern							
Live with family members	2542 (77.8)	2443 (79.5)	2661 (80.7)	2545 (81.1)	2592 (79.8)	12 783 (79.8)	.01
Live alone or at nursing home	724 (22.2)	629 (20.5)	638 (19.3)	592 (18.9)	656 (20.2)	3239 (20.2)	
Smoking practice							
Current smoker	533 (16.3)	561 (18.3)	731 (22.2)	656 (20.9)	666 (20.5)	3147 (19.6)	<.001
Former smoker	375 (11.5)	396 (12.9)	445 (13.5)	456 (14.5)	542 (16.7)	2214 (13.8)	
Nonsmoker	2358 (72.2)	2115 (68.8)	2123 (64.4)	2025 (64.6)	2040 (62.8)	10 661 (66.5)	
Alcohol consumption practice							
Current drinker	681 (20.9)	682 (22.2)	752 (22.8)	764 (24.4)	815 (25.1)	3694 (23.1)	<.001
Former drinker	288 (8.8)	284 (9.2)	323 (9.8)	322 (10.3)	360 (11.1)	1577 (9.8)	
Nondrinker	2297 (70.3)	2106 (68.6)	2224 (67.4)	2051 (65.4)	2073 (63.8)	10 751 (67.1)	
Regular exercise							
No	2131 (65.2)	1859 (60.5)	1909 (57.9)	1788 (57.0)	1700 (52.3)	9387 (58.6)	<.001
Yes	1135 (34.8)	1213 (39.5)	1390 (42.1)	1349 (43.0)	1548 (47.7)	6635 (41.4)	
Hypertension							
No	1690 (51.7)	1501 (48.9)	1539 (46.7)	1413 (45.0)	1314 (40.5)	7457 (46.5)	<.001
Yes	1576 (48.3)	1571 (51.1)	1760 (53.3)	1724 (55.0)	1934 (59.5)	8565 (53.5)	
Heart disease							
No	3109 (95.2)	2928 (95.3)	3127 (94.8)	2923 (93.2)	3011 (92.7)	15 098 (94.2)	<.001
Yes	157 (4.8)	144 (4.7)	172 (5.2)	214 (6.8)	237 (7.3)	924 (5.8)	
Type 2 diabetes							
No	3250 (99.5)	3055 (99.4)	3269 (99.1)	3098 (98.8)	3188 (98.2)	15 860 (99.0)	<.001
Yes	16 (0.5)	17 (0.6)	30 (0.9)	39 (1.2)	60 (1.8)	162 (1.0)	
Cerebrovascular disease							
No	3182 (97.4)	3008 (97.9)	3231 (97.9)	3057 (97.4)	3138 (96.6)	15 616 (97.5)	.005
Yes	84 (2.6)	64 (2.1)	68 (2.1)	80 (2.6)	110 (3.4)	406 (2.5)	
Respiratory disease							
No	2884 (88.3)	2712 (88.3)	2965 (89.9)	2802 (89.3)	2861 (88.1)	14 224 (88.8)	.09
Yes	382 (11.7)	360 (11.7)	334 (10.1)	335 (10.7)	387 (11.9)	1798 (11.2)	
Cognitive impairment							
No	2231 (68.3)	2288 (74.5)	2576 (78.1)	2569 (81.9)	2741 (84.4)	12 405 (77.4)	<.001
Yes	1035 (31.7)	784 (25.5)	723 (21.9)	568 (18.1)	507 (15.6)	3617 (22.6)	

^a^ Literacy was defined as receiving a formal education of more than 1 year; illiteracy was defined as receiving a formal education of less than 1 year.

**Figure 1.**
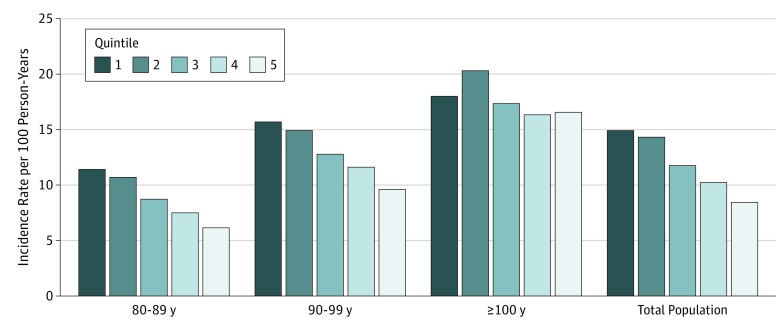
Incidence of Disability in Activities of Daily Living Among Participants With Different Body Mass Index Quintiles For the whole cohort, the incidence rate of disability in activities of daily living was 11.5 per 100 person-years, and was 14.9 per 100 person-years for the first quintile, 14.3 per 100 person-years for the second quintile, 11.7 per 100 person-years for the third quintile, 10.2 per 100 person-years for the fourth quintile, and 8.4 per 100 person-years for the fifth quintile.

Follow-up time was a mean (SD) of 4.4 (3.6) years (range, 0.1-16.3 years). During the 70 606 person-years of follow-up, 8113 participants (50.6%) with disability in ADL were identified. For the whole cohort, the incidence rate of disability in ADL was 11.5 per 100 person-years; for those 80 to 89 years of age, the incidence rate was 8.2 per 100 person-years; for those 90 to 99 years of age, the incidence rate was 12.6 per 100 person-years; and for those 100 years of age or older, the incidence rate was 17.8 per 100 person-years. For participants in the first quintile of BMI, the incidence rate of disability in ADL was 14.9 per 100 person-years; for those in the second quintile of BMI, the incidence rate was 14.3 per 100 person-years; for those in the third quintile of BMI, the incidence rate was 11.7 per 100 person-years; for those in the fourth quintile of BMI, the incidence rate was 10.2 per 100 person-years; and for those in the fifth quintile of BMI, the incidence rate was 8.4 per 100 person-years (Figure 1).

**Figure 2.**
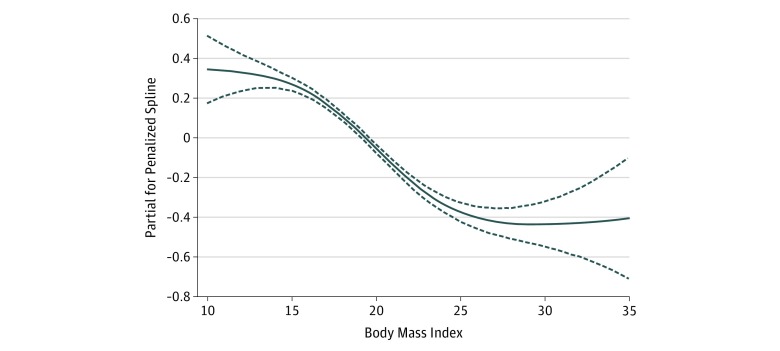
Association of Body Mass Index With Disability in Activities of Daily Living in Cox Proportional Hazards Regression Models With Penalized Splines The lines depict the estimated function of body mass index (calculated as weight in kilograms divided by height in meters squared) for risk of disability in activities of daily living (*df* = 3), and the dotted lines indicate the 95% CIs in the adults 80 years of age or older.

**Figure 3.**
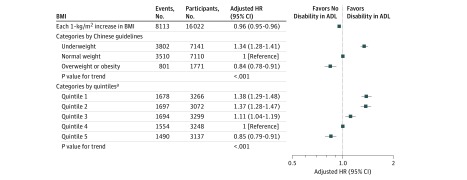
Associations of Body Mass Index (BMI) With Disability in Activities of Daily Living (ADL) in Adults 80 Years of Age or Older Adjusted for age, sex, residence, educational background, current marital status, living pattern, smoking practice, alcohol consumption practice, regular exercise, hypertension, diabetes, heart disease, cerebrovascular disease, and respiratory disease. HR indicates hazard ratio. ^a^The first BMI quintile was less than 16.2 (calculated as weight in kilograms divided by height in meters squared), the second quintile was from 16.2 to 17.9, the third quintile was from 18.0 to 19.8, the fourth quintile was from 19.9 to 22.1, and the fifth quintile was 22.2 or higher.

Cox proportional hazards regression models with penalized splines showed that BMI was linearly associated with risk of disability in ADL (*P* < .001) (Figure 2). Each 1-kg/m^2^ increase in BMI corresponded to a 4.5% decrease in risk of disability (HR, 0.955; 95% CI, 0.949-0.961). When BMI was categorized by quintiles, participants in the higher quintiles were less likely to develop disability: in comparison with individuals in the fourth quintile, the adjusted HR for disability in ADL was 1.38 (95% CI, 1.29-1.48) in the first quintile, was 1.37 (95% CI, 1.28-1.47) in the second quintile, was 1.11 (95% CI, 1.04-1.19) in the third quintile, and was 0.85 (95% CI, 0.79-0.91) in the fifth quintile (*P* < .001 for trend). When BMI was categorized by Chinese guidelines, the group categorized as underweight showed significantly increased risk of disability in ADL (HR, 1.34; 95% CI, 1.28-1.41) and the group categorized as overweight or obese showed a significantly decreased risk of disability in ADL (HR, 0.84; 95% CI, 0.78-0.91), compared with the group categorized as normal weight (*P* < .001 for trend) (Figure 3).

**Figure 4.**
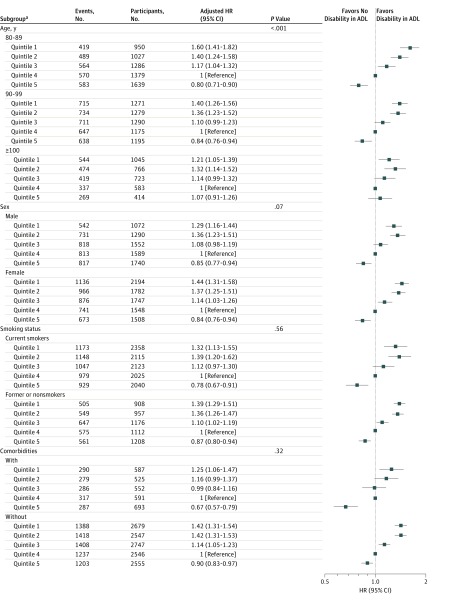
Subgroup Analyses of Associations of Body Mass Index With Disability in Activities of Daily Living (ADL) in Adults 80 Years of Age or Older Adjusted for age, sex, residence, educational background, current marital status, living pattern, smoking practice, alcohol consumption practice, regular exercise, hypertension, diabetes, heart disease, cerebrovascular disease, and respiratory disease. HR indicates hazard ratio. ^a^The first body mass index quintile was less than 16.2 (calculated as weight in kilograms divided by height in meters squared), the second quintile was from 16.2 to 17.9, the third quintile was from 18.0 to 19.8, the fourth quintile was from 19.9 to 22.1, and the fifth quintile was 22.2 or higher.

There was a significant interaction of BMI with age for risk of incident disability in ADL. This finding suggested that the reverse association was modified by age; it was more prominent in those 80 to 89 years of age and in those 90 to 99 years of age than in those 100 years of age or older. In addition, there were no significant interactions of sex, smoking status, or comorbidity with BMI for risk of incident disability in ADL. These estimated effects were similar in those subgroups, and the reverse associations were robust in men and women, smokers and nonsmokers, and participants with and without identified comorbidities (Figure 4).

## Discussion

In the largest cohort study, to our knowledge, in this field among Chinese adults age 80 years or older, we observed prospective longitudinal associations of objectively measured BMI with incidence of disability in ADL in individuals 80 to 89 years of age, 90 to 99 years of age, or 100 years of age or older. Among the total cohort of adults 80 years of age or older and in each of the 3 age groups, the participants with higher BMIs had lower risk of disability in ADL than did those with lower BMIs.

The influence of age on the association of BMI with physical functioning has been a subject of much debate. For middle-aged individuals, U-shaped or J-shaped associations were found between BMI and physical functioning.^27^ With regard to BMI in older adults, previous studies were controversial concerning whether overweight increased the risk for disability in ADL^9,15^ or even had protective effects^28,29^; one study concluded that the cutoff point of a BMI of 25 or higher for overweight might overestimate the risks for older people and that this threshold should be raised, as BMI levels between 25.0 and 29.0 may not be associated with excess risk in the older adults.^30^ However, in one meta-analysis,^31^ overweight was associated with higher risk of disability in ADL in both cross-sectional and longitudinal studies.

Regarding adults 80 years of age or older, although 2 cross-sectional studies have shown that obesity had an adverse association with physical functioning,^16,17^ longitudinal studies, including our present study, have shown that participants with overweight or obesity were likely to have better functional performance.^18,19^ In a cross-sectional study with 870 Chinese long-lived adults (those 90-99 years of age and those ≥100 years of age), the risk of disability in ADL and instrumental ADL was higher for women with both extremely low and high BMI, but the finding was not seen in men.^15^ Similarly, in a previous cross-sectional study based on the CLHLS,^17^ J-shaped associations of BMI with disability in ADL were revealed, and differed between the sexes. The cutoff points of BMI for lowest risk of disability in ADL were 18.5 for men and 20.0 for women. In contrast, and consistent with our findings, longitudinal studies^18,19^ showed that higher BMI, even those indicating overweight or obesity as defined by guidelines, was associated with lower risk of disability in ADL in those age 80 years or older. The most possible reason for the inconsistencies is the study design. The cross-sectional study measures the BMI and disability in ADL simultaneously; it fails to fully control for individual baseline values, and does not permit distinction between cause and effect. Although a prospective cohort study measures exposures and events in chronological order and the participants with baseline disability in ADL were excluded, it can be used to distinguish between cause and effect. Thus, the present study is theoretically more powerful and provides a more precise risk estimation of the association of BMI with disability in ADL than did the previous cross-sectional study.

It is well recognized that an indirect measure of anthropometry, such as BMI, is a measure of muscle mass rather than body fatness in older adults because of the change in skeletal muscle and abdominal fat with aging.^32^ Perhaps higher BMI is a marker of obesity-associated vascular disease that predominates the risk equation before 80 years of age; this hypothesis was partly supported by our results that higher BMI increased the odds of hypertension, diabetes, heart disease, stroke, and cerebrovascular disease in this study. However, after 80 years of age, declining and low BMI may denote a predominance of malnutrition or muscle mass attenuation, which then results in a decrease in physical strength and more severely affected function as a marker for disability.^33^ Body mass index is often used as an indicator of nutritional status and even as an indicator of health status. Underweight due to malnutrition or sarcopenia might also lead to disability through fewer social interactions and less activity, thereby leading to a higher risk of bone loss, falls, and fractures.^34^

Body weight loss or underweight (defined as a BMI <18.5) has been considered one of the most important components of the frailty index.^35^ A multicenter study of community-dwelling individuals revealed that almost all components of the frailty index were associated with disability in ADL and instrumental ADL (transportation, shopping, housekeeping, food purchasing, and food preparation) in older adults.^36^ There is also evidence that low body weight can be associated with a lack of physical activity owing to difficulties in performing certain movements and an increase of sedentariness.^5^ Disability may also be a cause of underweight through difficulties in eating, obtaining provisions, and cooking, which can be reflected by disability on the items of the ADL, while the reverse causality did not likely occur owing to the prospective study design.

A major concern of this study was the potential survival bias or selection bias generated by the composition of the sample (ie, individuals who survived to ≥80 years of age). Although the association of BMI with disability in ADL was strong in this study, it could have been overestimated. Being overweight or obese was linked to a higher risk of mortality and disabling pathologic problems before the age of 80 years. As a consequence, the participants with overweight or obesity might be excessively selected as a result of still being alive, healthy enough to live to 80 years of age or older, and simultaneously have normal ADL to meet the inclusion criteria of this study. Among the participants in this study, 44.6% of the men were underweight and 11.1% were overweight or obese. The mean BMI of the participants was 19.3, which was similar to a previous study that showed that the mean BMI of Chinese individuals 90 to 99 years of age or 100 years of age or older was 19.2,^15^ but was much lower than the BMI reported for the general adults. The inverse association of BMI and disability in ADL was not greatly affected by confounders (including poor health and smoking status, which produce lower BMI and increased disability risk) or by performing subgroup analysis in the healthy group without identified comorbidities or in former smokers or nonsmokers. This finding suggested that selection bias may be, in part, an explanatory factor, but is not likely to be the main explanatory factor of the finding.

Taken together, causation of the inverse association between BMI and disability in ADL is probably multifactorial. Malnutrition, frailty, survival bias or selection bias, and reverse causation were considered to be potential explanations. The study included as many as 16 022 community-based participants with normal ADL, the largest sample size of its kind, which allowed us to explore the association among men and women and among individuals 80 to 89 years of age, 90 to 99 years of age, and 100 years of age or older. In addition, several identified confounders of disability in ADL with BMI were controlled; sensitivity analyses or subgroup analyses were also performed to explore reverse casualty and selection bias, which made our finding more confident. Strengths of this study included objective anthropometric measurements and functional status assessed by ADL scales.

### Limitations

This study should be interpreted considering several limitations. First, owing to the high prevalence of osteoporosis and/or kyphosis in the adults age 80 years or older, the calculated BMI may have been overestimated. Owing to the low prevalence of obesity among adults 80 years of age or older, a clear conclusion regarding the association of the conventional BMI for obesity with disability in ADL is not likely to be drawn. Second, BMI in our study was closely correlated with some baseline characteristics, such as smoking practice, alcohol consumption practice, and regular exercise, which in turn are likely to be closely associated with incidence of disability in ADL. Although we carefully controlled for numerous potential confounders and obtained similar results, residual confounding is still possible owing to the observational study design and the absence of quantitative assessment on certain confounders. Third, owing to excluding 5019 participants lost to follow-up at the first follow-up survey, selection bias may confound the finding, even though the differences between the included and excluded participants were very small. Fourth, the event time to disability in ADL was defined as the period from baseline to the first time a participant experienced disability; however, the participants could be disabled any time during a 2- to 3-year interval, rather than the exact date of the event, which may bias the present finding. Fifth, this study focused on a specific population, and the BMI of Chinese adults 80 years of age or older is generally lower than other ethnic groups and other younger age groups; thus, the present finding should be prudently applied to other ethnic and age groups.

## Conclusions

Higher BMI was associated with a lower risk of disability in ADL for adults 80 years of age or older, which lends further support to the opinion that guidelines recommended by World Health Organization or the Working Group on Obesity in China for overweight and obese individuals do not apply to this age group. Thus, more attention should be paid to the issue of being underweight rather than overweight or obese after 80 years of age for the prevention of disability in ADL. With an increasing life expectancy and an increase in the population prevalence of overweight and obesity, it is important to reevaluate the role of BMI in the population burden of disability in the future among the adults 80 years of age or older.

## Notes

Supplement. **eAppendix.** Testing the Proportional Hazard Assumption **eFigure 1.** Flowchart of the Participants’ Inclusion **eFigure 2.** Testing the Proportional Hazard Assumption With Kaplan-Meier Curves **eFigure 3.** Testing the Proportional Hazard Assumption Based on the Schoenfeld Residuals **eFigure 4.** The Association Between the Schoenfeld Residuals of BMI and the Function of Time
