# Supplementary material for: Association of Body Mass Index With Disability in Activities of Daily Living Among Chinese Adults 80 Years of Age or Older
Source: JAMA Netw Open. 2018 Sep 7;1(5):e181915. doi: 10.1001/jamanetworkopen.2018.1915 (PMC6324469; doi:10.1001/jamanetworkopen.2018.1915)
Supplement: Supplement. — eAppendix. Testing the Proportional Hazard Assumption eFigure 1. Flowchart of the Participants’ Inclusion eFigure 2. Testing the Proportional Hazard Assumption With Kaplan-Meier Curves eFigure 3. Testing the Proportional Hazard Assumption Based on the Schoenfeld Residuals eFigure 4. The Association Between the Schoenfeld Residuals of BMI and the Function of Time [file jamanetwopen-1-e181915-s001.pdf]

Supplementary Online Content

Lv Y-B, Yuan J-Q, Mao C, et al. Association of body mass index with disability in activities of daily living among Chinese adults 80 years of age or older. *JAMA Netw Open.* 2018;1(5):e181915. doi:10.1001/jamanetworkopen.2018.1915

eAppendix. Testing the Proportional Hazard Assumption
eFigure 1. Flowchart of the Participants' Inclusion
eFigure 2. Testing the Proportional Hazard Assumption With Kaplan-Meier Curves
eFigure 3. Testing the Proportional Hazard Assumption Based on the Schoenfeld Residuals
eFigure 4. The Association Between the Schoenfeld Residuals of BMI and the Function of Time

This supplementary material has been provided by the authors to give readers additional information about their work.

© 2018 Lv Y-B et al. *JAMA Network Open.*

eFigure 1. Flowchart of the Participants' Inclusion

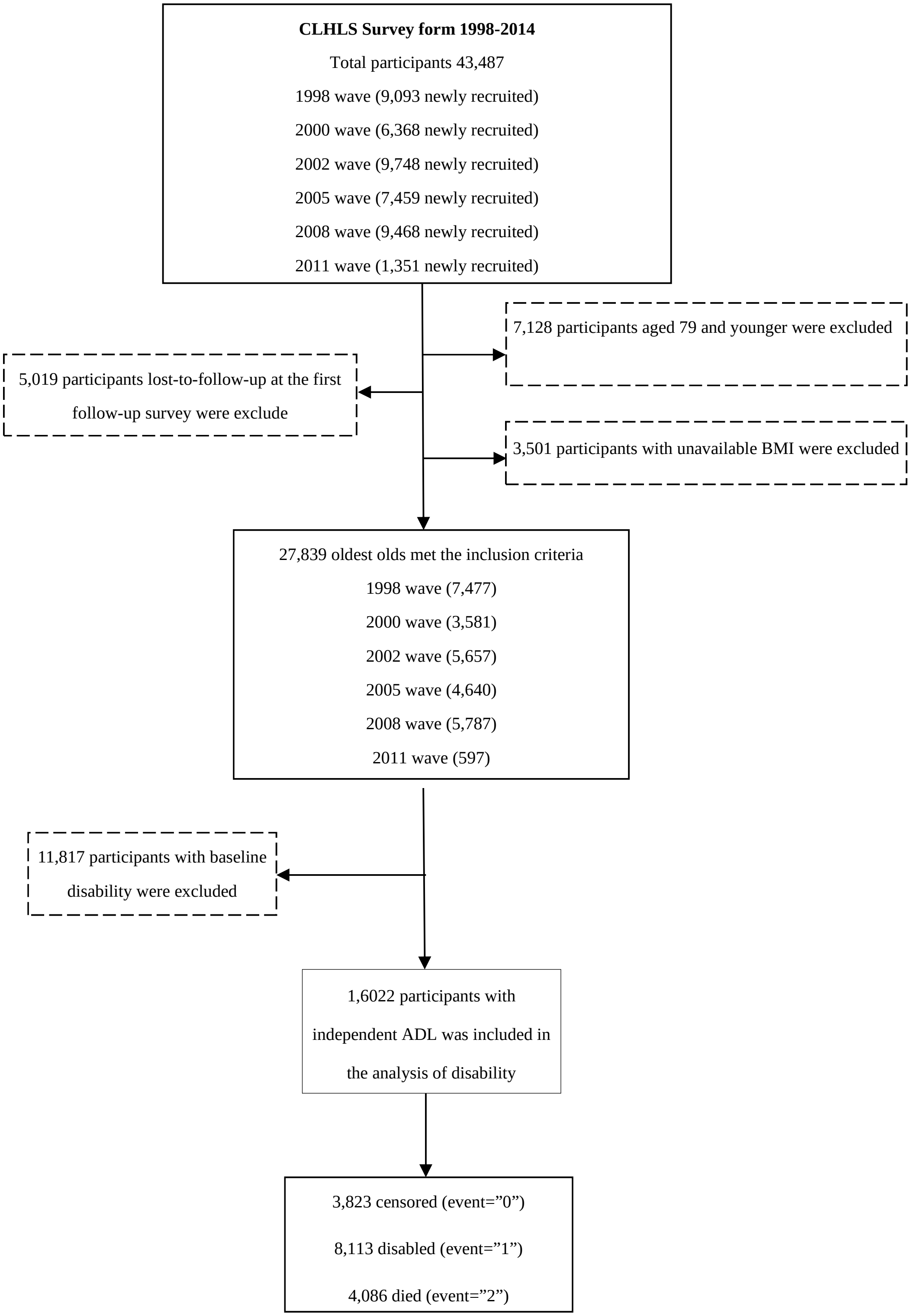

© 2018 Lv Y-B et al. *JAMA Network Open.*

eAppendix. Testing the Proportional Hazard Assumption

Proportional hazard assumption was tested with Kaplan-Meier curves when BMI was taken as categorized variable, then was tested by the linear regression of the scaled Schoenfeld residuals on functions of time when BMI was taken as continuous variable. It showed that the assumption of proportional hazard assumption was satisfied.

In the Kaplan-Meier curves, the graph of the "survival function" versus the "survival time" results in a graph with parallel curves, which suggests the quintiles of BMI satisfy the proportional hazard assumption (eFigure 2) .

© 2018 Lv Y-B et al. *JAMA Network Open.*

eFigure 2. Testing the Proportional Hazard Assumption With Kaplan-Meier Curves

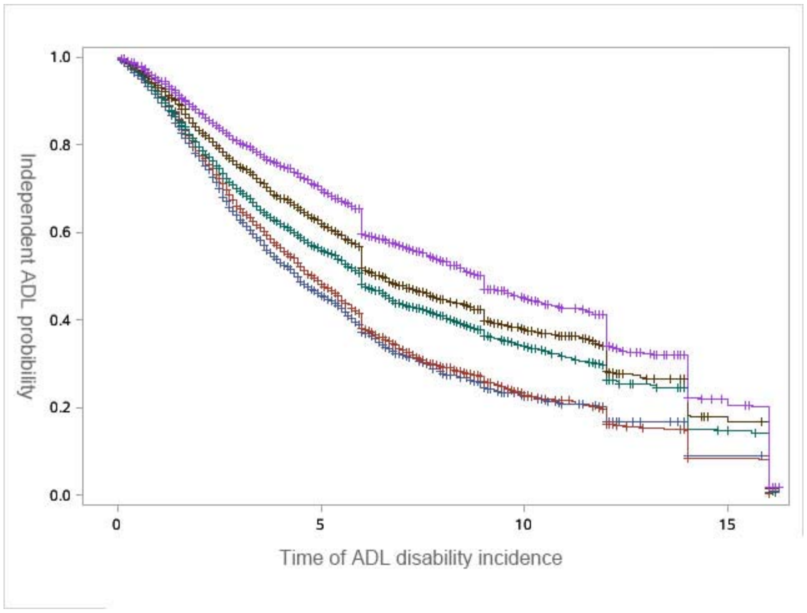

The Kaplan-Meier curves method does not work well for continuous predictor or categorical predictors that have many levels because the graph becomes to "cluttered". To test whether the proportional hazard assumption in models were met when BMI was taken as continuous variable, the Schoenfeld Residuals were calculated (Schoenfeld D. Partial residuals for the proportional hazards regression model. *Biometrika*, 1982, 69(1):239-241.). (eFigure 3) The Schoenfeld residual is defined as the covariate value for the individual that failed minus its expected value:

$$\text{residual} = x_{ik} - \sum_{i=1}^{j \in R(t_i)} x_{jk} p_j$$

© 2018 Lv Y-B et al. *JAMA Network Open.*

eFigure 3. Testing the Proportional Hazard Assumption Based on the Schoenfeld Residuals

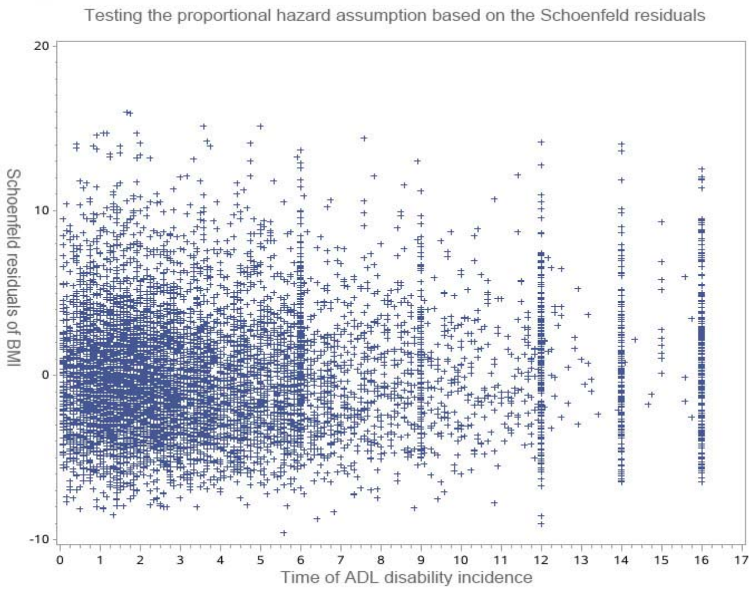

Testing the time dependent covariates is equivalent to testing for a non-zero slope in a linear regression of the scaled Schoenfeld residuals on functions of time. It showed that the slope of the linear regression is 0.010 (R-square of the model is 0.004), it was taken as meeting the proportional hazard assumption regard to the weak relationship of the residuals with the function of time. (eFigure 4)

© 2018 Lv Y-B et al. *JAMA Network Open.*

eFigure 4. The Association Between the Schoenfeld Residuals of BMI and the Function of Time

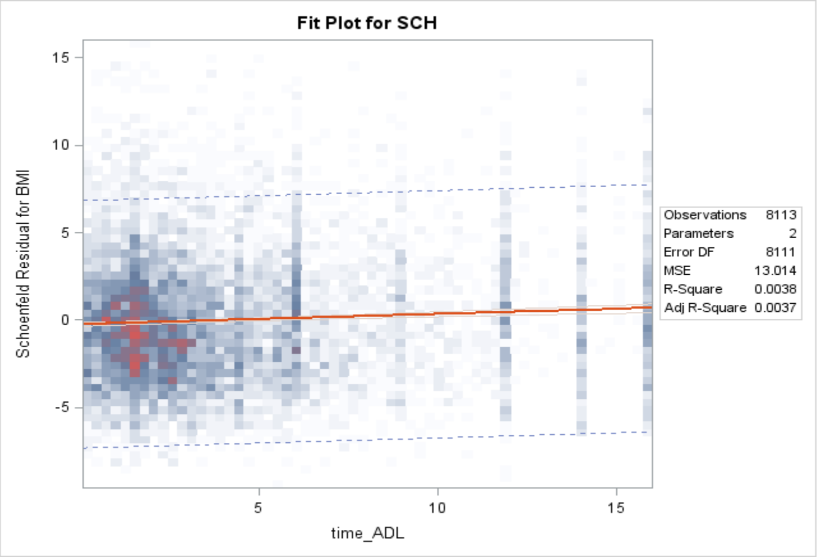

© 2018 Lv Y-B et al. *JAMA Network Open.*
